# Effect of the Drying Method and Storage Conditions on the Quality and Content of Selected Bioactive Compounds of Green Legume Vegetables

**DOI:** 10.3390/molecules29081732

**Published:** 2024-04-11

**Authors:** Piotr Gębczyński, Małgorzata Tabaszewska, Katarzyna Kur, Maria Zbylut-Górska, Jacek Słupski

**Affiliations:** 1 Department of Plant Product Technology and Nutrition Hygiene, Faculty of Food Technology, University of Agriculture in Krakow, Al. Mickiewicza 21, 31-120 Krakow, Poland; piotr.gebczynski@urk.edu.pl (P.G.); katarzyna.kur@poczta.fm (K.K.); jacek.slupski@urk.edu.pl (J.S.); 2 Department of Land Surveying, Faculty of Environmental Engineering and Land Surveying, University of Agriculture in Krakow, Al. Mickiewicza 21, 31-120 Krakow, Poland; maria.zbylut-gorska@urk.edu.pl

**Keywords:** green legume, drying, storage period, storage conditions, bioactive compounds

## Abstract

This study aimed to determine the effect of the drying method (freeze-drying, air-drying), storage period (12 months), and storage conditions (2–4 °C, 18–22 °C) applied to two legume species: green beans and green peas. The raw and dried materials were determined for selected physical parameters typical of dried vegetables, contents of bioactive components (vitamin C and E, total chlorophyll, total carotenoids, β-carotene, and total polyphenols), antioxidative activity against the DPPH radical, and sensory attributes (overall quality and profiles of color, texture, and palatability). Green beans had a significantly higher content of bioactive components compared to peas. Freeze-drying and cold storage conditions facilitated better retention of these compounds, i.e., by 9–39% and 3–11%, respectively. After 12 months of storage, higher retention of bioactive components, except for total chlorophyll, was determined in peas regardless of the drying method, i.e., by 38–75% in the freeze-dried product and 30–77% in the air-dried product, compared to the raw material.

## 1. Introduction

The Fabaceae is the third largest plant family, including about 5000 edible species [1]. Approximately 100 Fabaceae species have been recorded in Poland, the edible varieties of which are represented by, among others, beans and peas (both ranked among the three major crops grown in Poland), broad beans, lentils, soybeans, and chickpeas [2]. Dry (ripe) seeds of legumes are most often used for culinary purposes as a stable and easy-to-store raw material. Nonetheless, legumes are also consumed when not completely ripe; e.g., their whole pods feature a different composition and characteristics [3] and are especially valued for their significantly higher contents of biologically-active compounds, like vitamins (C, E), pigments (chlorophylls and carotenoids in particular), and polyphenols (phenylpropanoids, flavonoids, isoflavones, catechins, tannins, coumarins, furanocoumarins, phenolic acids) compared to dry seeds [4,5,6,7]. These compounds are essential to the human body as they are capable of inhibiting or arresting oxidation processes and compensating for the effects of free radicals that cause damage to cells, leading to lesions in the body and development of many lifestyle-related, non-communicable diseases [8,9]. In addition, as fine sources of plant-derived protein, pulses have become a basic element of vegetarian and vegan diets, which have recently spurred huge consumer interest. The United Nations (UN) has paid special attention to legumes as a source of staple food across the globe and has established the World Pulses Day [10].

Vegetables, including legumes, play a very important role in human nutrition, as they provide components regulating biological processes in the human body, thereby strongly contributing to the maintenance of good health status. Fruits and vegetables are essential constituents of a healthy and well-balanced diet [11,12], represent the core element of the Healthy Nutrition and Physical Activity Pyramid established by the State Institute of Public Health in Poland, and they should be consumed every day in appropriate amounts and coupled with physical activity [13,14].

Freshly harvested vegetables are unstable in terms of their quality and storage potential. Their stability may be extended, and the abundance of their nutrients may be retained through preservation [15]. The oldest methods deployed for stability extension include drying, which reduces product weight and, depending on the method applied, facilitates the preservation of taste, aroma, and high contents of bioactive compounds [16]. Dried vegetables may be used as semi-products to manufacture ready-to-eat foods, seasoning mixtures, and snacks [17]. They are convenient to transport and store, easily distributed across the globe, and can even be taken into the cosmic space. The drying method, conditions, and storage period often determine the physical and sensory traits and also the retention of bioactive components in dried products [18]. The available literature provides no reports on the impact of conditions similar to those applied in the present study and long-term storage (12 months) of air-dried and freeze-dried materials from legumes (green beans, green peas) on the analyzed quality attributes of the finished products. In addition, the present study investigated the storage conditions of dried materials applied in households, gastronomy, and in retail. Although there is literature devoted to the impact of various packages on the stability and quality of dried fruits, only a few of them concern everyday use conditions.

## 2. Results and Discussion

### 2.1. Effect of Blanching on the Analyzed Parameters

Vegetables are sources of many valuable nutrients and bioactive compounds, which may be affected by the technological process, including raw material treatment. Fresh green beans had a significantly higher water content, were darker, and had a lesser contribution of yellow color in the color profile compared to fresh peas. In addition, they featured significantly higher contents of the analyzed bioactive compounds, except for vitamin E, a higher content of which was determined in fresh peas. The blanching of the fresh vegetables significantly impacted changes in their color parameters and decreased the contents of vitamins and total chlorophyll. It reduced their antioxidative activity compared to the non-blanched materials (Table 1).

The variance analysis demonstrated that blanching green beans caused significant darkening (as indicated by a decreased *L* value), reduced green color contribution in the color profile, vitamin C content, total chlorophyll content, and antioxidative activity, and increased the total polyphenol content. In turn, blanching green peas had a less pronounced effect on the analyzed parameters (Supplementary Figure S1).

Color darkening, caused by a decrease in *L* parameter values, may be explained by the fact that blanching removes air from the plant tissue and causes it to shrink, intensifying the initial color [19], especially in non-white materials. Blanching elicited statistically significant changes in the vitamin C content of the plant material. Vitamin C is poorly stabile in hydrothermal processes; hence, it is important for the blanching to be as short as possible to minimize vitamin C diffusion to water and its thermal degradation. The above-presented results are consistent with findings reported earlier by Gębczyński and Lisiewska [20], Gębczyński and Kmiecik [21], Sikora et al. [22], Volden et al. [23], as well as Lee and Choo [24]. In contrast, opposite results were reported for green beans by Baardseth et al. [25], who showed that no vitamin C losses occurred during blanching. In turn, high losses (40%) of vitamin E in blanched green beans were demonstrated by Piotrowski et al. [26]. Their findings are inconsistent with the results of the present study, which shows no significant effect of blanching on vitamin E content. Many authors have noted a significant effect of blanching on the content of carotenoids [27,28,29,30]. In contrast, Gębczyński and Kmiecik [21] showed no effect of this process on the carotenoid content of cauliflower, which agrees with our observations in this respect. The present study also showed no significant effect of blanching on the content of β-carotene. Opposite findings were reported by Wen et al. [31], who noted a 31%, 102%, 106%, and 5% increase in goa beans, green beans, long beans, and sugar snap peas, respectively, after their so-called blanching (cooking in water for 10 min). Like in the present study, Negi and Roy [32] demonstrated 42%, 26%, and 14% losses of chlorophylls in water-blanched beetroot, amaranth, and fenugreek, respectively. Compared to the present study, greater losses of phenolic compounds in fresh peas (25%) and green snap peas (45%), as well as their increased contents in green beans (by 32%) and goa beans (74%), were demonstrated by Wen et al. [31]. Thermal processes, like blanching or drying, may trigger both losses and increases in the contents of polyphenols. In plants, phenolic compounds are bound with dietary fiber, proteins, and sugars. The high-temperature treatment causes material tissue to soften. It increases extraction from the matrix and generates secondary plant metabolites, which exhibit higher antioxidative activity than the native compounds [33,34]. The blanching of raw materials in the present study resulted in statistically significantly suppressed antioxidative activity of the vegetables, which is consistent with findings reported by other authors [18,21,31,35].

### 2.2. Effect of Drying and Storage on the Analyzed Parameters

#### 2.2.1. Physical Properties

**Figure 1:**
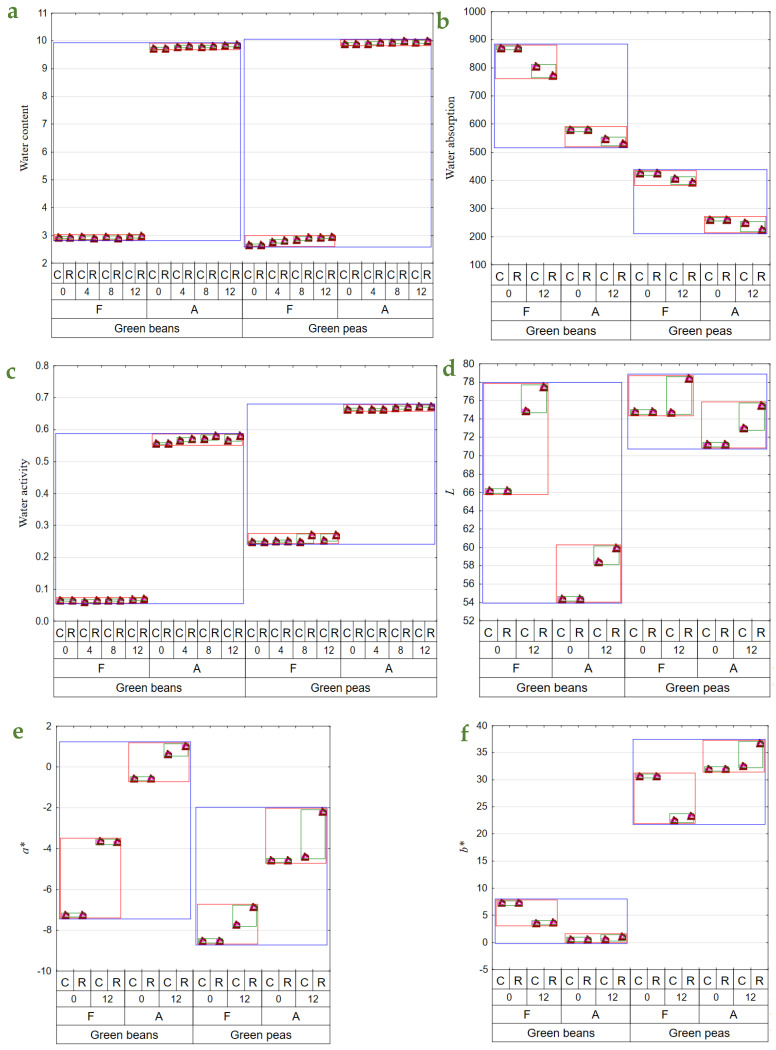

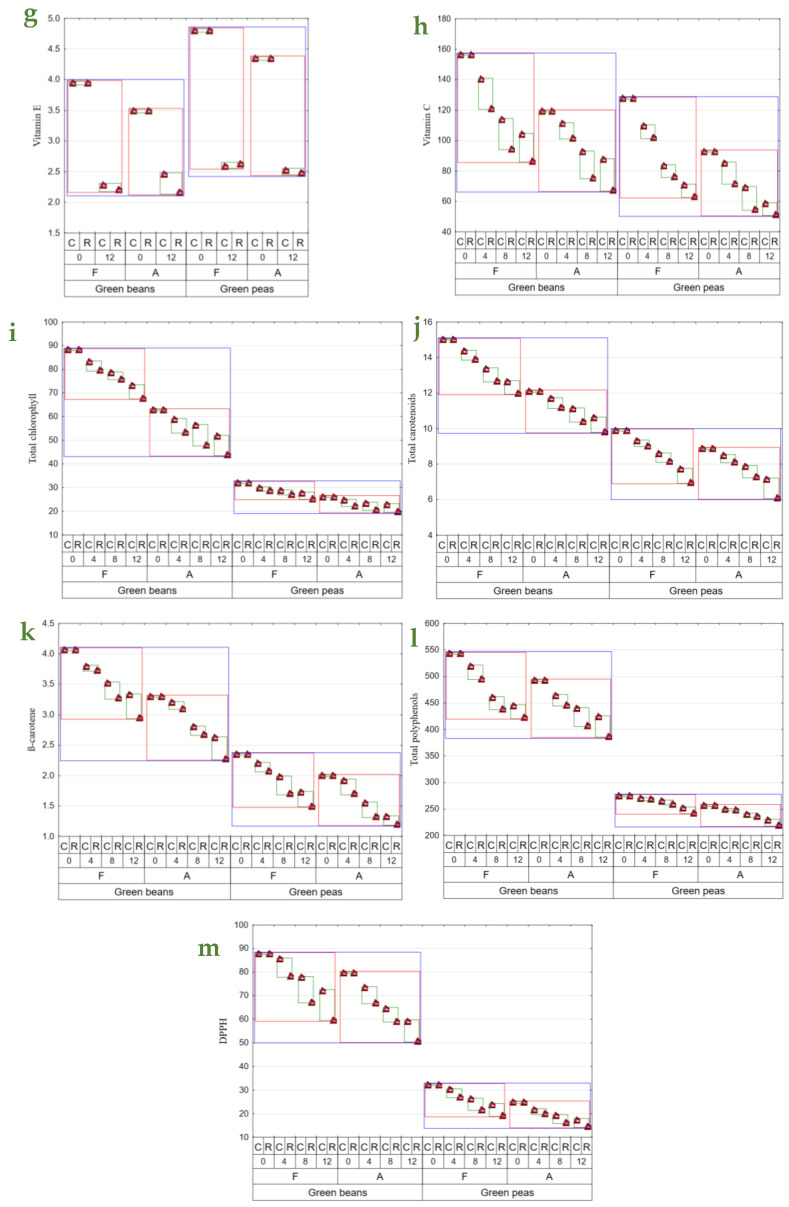
Mean values of: (**a**)—water content (g × 100 g d.f.^−1^); (**b**)—water absorption (g × 100 g d.w.^−1^); (**c**)—a_w_; (**d**)—color parameter *L*; (**e**)—color parameter a*; (**f**)—color parameter b*); (**g**)—vitamin E content (mg × 100 g d.w.^−1^); (**h**)—vitamin C content (mg × 100 g d.w.^−1^); (**i**)—total chlorophyll content (mg × 100 g d.w.^−1^); (**j**)—total carotenoid content (mg × 100 g d.w.^−1^); (**k**)—β—carotene content (mg × 100 g d.w.^−1^); (**l**)—total polyphenol content (mg (+)catechin × 100 g d.w.^−1^); (**m**)—antioxidant activity against a radical DPPH (μmol Trolox equivalent × g d.w.^−1^) in vegetable green beans and green peas (blue line), depending on the method of drying (F—freeze-drying; A—air-drying; red line), storage period (0, 4, 8, 12 months), and temperature of storage (C—2–4 °C; R—18–22 °C; green line).

The drying and storage of dried materials under various conditions affected their physical features, contents of bioactive components, and perceptible sensory impressions [16]. Dried green beans had substantially higher contents of the determined bioactive components and, at the same, exhibited considerably lower water activity. The freeze-dried vegetables had significantly higher contents of bioactive components and significantly lower water content and activity. The contents of bioactive components decrease substantially over the storage period, with significantly greater losses noted in the dried materials stored at room temperature (Table 2 and Table 3). Also, during storage, a slight increase in water content (to 2%) in BF12R was noted. A greater water content increase was recorded during PFR and C storage, with the greatest increase in water content determined in PF12R (Figure 1a). In both types of air-dried materials (beans and peas), ca. 1% increase was determined in water content over the entire storage period, compared to the initial value. This increase could be due to moisture absorption from the environment when opening jars with dried materials while collecting samples for analyses. The freeze-dried materials have a more porous structure and are easier to hydrate. Given the long period of raw material exposure to high temperatures, air drying caused permanent changes in the material’s structure, thereby reducing its capability for water re-absorption. In turn, due to the process specificity, freeze-drying results in a highly porous final product exhibiting a higher rehydration capability [36,37]. When analyzed immediately after drying, the freeze-dried vegetables exhibited a higher water absorption capacity than those obtained upon air drying (Figure 1b), consistent with other authors’ findings [36,38]. The freeze-dried pulses showed 50–62% better water absorption capacity than their air-dried counterparts. After 12 months of storage, the water absorption capacity of the freeze-dried materials decreased by 4–15% compared to the vegetables determined immediately after drying (Figure 1b).

Water activity determined over the storage period changed only slightly in BF, whereas it was observed to increase successively in BA, especially in the dried materials stored at room temperature (ca. 5% increase). In the case of PF, water activity also increased, especially in the samples stored at room temperature for 12 months (an increase to ca. 11% after 12 months of storage). In contrast, the changes in water activity of PA were small throughout the entire storage period (Figure 1c). The reduced water content and, consequently, water activity affect dried materials in terms of their microbiological quality as well as chemical and physical transformations. The minimal a_w_ required for developing bacteria, yeast, and molds reached 0.9, 0.8, and 0.7, respectively. Chemical reactions entail enzymatic transformations, oxidation of food constituents, and reactions of non-enzymatic browning (NEB). The water content affects the conformation of enzymatic proteins, which determine enzyme activity and catalytic capability. The action of enzymes such as amylases, phenoloxidases, or peroxidases is inhibited at a_w_ ≤ 0.8. In turn, lipids’ oxidation proceeds in a dry environments with deficient water activity, i.e., a_w_ 0.1–0.3. Similar transformations may be observed for fat-soluble compounds, like carotenoids, or certain aroma compounds. In turn, the oxidation rate of water-soluble substances, like vitamin C, heme pigments, and chlorophylls, increases along with increasing a_w_. NEB reaction intensity also increases with a_w_ increase and reaches the maximum at 0.3–0.7, depending on product type [39,40]. Shukla and Singh [41] demonstrated a_w_ = 0.75 in cauliflower, champignon, and green peas dried by freeze-drying coupled with convection drying.

There were no changes in *L* parameter values in PFC and PAC (Figure 1d). The same observation was made by Michalik and Dobrzański [42] during a 3-month storage of air-dried dill, celery, and parsley leaves. In addition, a statistically significant color brightening was demonstrated after the 12-month storage for freeze-dried and air-dried green beans. Immediately after drying, the freeze-dried materials had a lighter color than the air-dried ones. Cao et al. [43] noted similar changes in air-dried broccoli. Storage under room conditions facilitated greater color brightening of the dried materials.

Air-drying caused a lesser contribution of green in the color profile than freeze-drying. During the storage of dried beans, the contribution of green was observed to decrease, regardless of the drying method, and was also slightly affected by storage temperature. In turn, the storage of peas also resulted in a lesser contribution of green in the color profile, except for PAC, which did not exhibit any significant changes in a* parameter values (Figure 1e). The storage of dried peas at ambient temperature reduced green color contribution in the color profile, regardless of the drying method. In turn, Cao et al. [43] reported the preservation of a more intense green color in freeze-dried broccoli than in air-dried broccoli.

Yellow made a greater contribution to the color profile over the entire storage period for dried materials made of peas. The b* parameter value decreased in the freeze-dried materials during storage, regardless of temperature. In contrast, changes observed in this parameter in the air-dried materials were small, except for PA12R, in which a greater contribution of yellow was noted compared to the cold-stored dried materials (Figure 1f). An increase in the b* parameter value was also noted in air-dried peas [44] as well as celery and parsley [45]. El-Hamzy and Ashour [38] reported an increase in b* parameter values during the storage of dried bell peppers at room temperature, regardless of the drying method applied.

#### 2.2.2. Contents of Bioactive Components

The study showed no significant effect of storage temperature on vitamin E retention. An over 40% decrease was noted in vitamin E content throughout the experimental period, except for BA, whose losses were below 40%. The storage of green beans at room conditions contributed to greater losses of this vitamin compared to peas, in which its losses were small (Figure 1g). Also, Syamila et al. [46] demonstrated significant losses of α-tocopherol in spinach already after 2 months of storage; however, in the case of juice, its extraction from cell structure certainly accelerated enzymatic and non-enzymatic transformations. The content of vitamin C decreased over the study period in both beans and peas, regardless of drying method and storage conditions; however, its retention was lower during storage at room conditions and also in the air-dried materials (Figure 1h). In BFC, vitamin C content decreased by 10% to 33% after 4 and 12 months of storage, respectively, compared to its content determined in the dried materials immediately after production, as well as from 22% to 44% in the materials stored at room conditions. Vitamin C losses determined in BA were similar to those noted in the freeze-dried beans. Its losses in PFC were greater than in beans, i.e., by 4% to 46% after 4 and 12 months of storage, respectively, and by 20% to 51% in the dried materials stored at room conditions. The losses of vitamin C determined in PA were usually smaller than those noted in PF. Qing-Guo et al. [47] observed dried green soybean pods similarly, as they reported comparable and greater vitamin C losses for freeze-dried and air-dried pulses, respectively. Vitamin C is susceptible to oxygen and high temperature; hence, reducing its impact during drying is essential [48,49]. A comparison of dried materials obtained with the two analyzed drying methods revealed that the freeze-dried ones had a higher vitamin C content than the air-dried ones. This result was due to the nature of the freeze-drying process, during which rehydrated material is placed in a hermetically sealed chamber under reduced pressure, and the highest temperature of its treatment reached 30 °C during re-drying. The dried material was exposed to hot air with temperatures of 50–60 °C in the air-drying method. Similar conclusions were drawn by Korus [18] and Marić et al. [45] in their studies with kale, dill, yellow carrot, and parsley, where freeze-drying allowed for 10% to 75% higher retention of vitamin C compared to air-drying. Previous investigations have emphasized the role of storage temperature in vitamin C retention and also the fact that its increase facilitates vitamin degradation [18,50,51]. Korus [18], who analyzed kale samples subjected to freeze-drying and air-drying, stored for 12 months in glass jars at room temperature (18–20 °C) and under cold storage conditions (8–10 °C), reported 12% and 15% higher vitamin C retention in the cold-stored samples, respectively. In addition, vitamin C losses were greater in the non-blanched material.

The total chlorophyll content was lower during the entire study period in the air-dried materials, i.e., from 44 to 63 mg × 100 g d.w.^−1^ in beans and from 20 to 26 mg × 100 g d.w.^−1^ in peas, compared to the freeze-dried samples, i.e., from 67 to 88 mg × 100 g d.w.^−1^ in beans and from 25 to 32 mg × 100 g d.w.^−1^ in peas. During storage, it was observed to decrease, yet to a greater extent in the dried vegetables stored at room temperature conditions. The changes observed throughout the experiment in the content of total chlorophyll in dried peas were usually smaller than those noted in beans (Figure 1i). Higher chlorophyll retention after freeze-drying than air-drying was also reported by Cui et al. [52] and Michalik and Dobrzański [42]. The cold storage conditions applied to the dried vegetables also enabled 8% higher chlorophyll retention in the freeze-dried pulses. In the case of air-drying, higher retention of chlorophyll was demonstrated in beans (by 18%) and in peas (by 15%) kept under cold storage conditions compared to room temperature conditions. Most investigations addressing the storage of chlorophyll-rich dried materials refer to the storage periods of 3 months [51,53,54] and 9 months [50]. They often concern only one of the drying methods or one of the storage temperatures analyzed in the present study, and they usually investigate different temperature ranges. For this reason, it is not easy to compare the results from the present study with those achieved by the authors above. Depending on the package used, Seevaratnam et al. [54] reported 4–11% chlorophyll losses in air-dried Brazilian spinach and amaranth stored at room temperature (28–36 °C). Also, depending on the package used, Singh and Sagar [51] demonstrated 14–30% chlorophyll losses in air-dried curry stored at room temperature (15–35 °C) and 8–20% losses in the cold-stored curry samples (7 °C), as well as 19–40% and 14–25% chlorophyll losses in air-dried moringa samples stored under respective temperatures.

Air-drying caused a greater decrease in the total carotenoid content in dried vegetables (especially in beans) than freeze-drying. The content of total carotenoids decreased successively throughout the experiment, but still, their losses were greater in the samples stored at room temperature than in the cold-stored ones. During the 12-month storage, the total carotenoid losses reached 16% in BFC, 20% in BFR, and 12% in 19% of the respective air-dried samples. In this storage period, greater carotenoid losses were recorded in PFC (22%) and PFR (30%). In contrast, the losses determined in PA reached 19% and 31%, respectively (Figure 1j). Cui et al. [52] and Cui et al. [36] noted 29% losses of the total carotenoids in the air-dried samples and significantly lesser losses (5%) in the samples subjected to the microwave–vacuum treatment. Nowacka et al. [55] also reported that storage period and temperature had a statistically significant effect on the reduced content of total carotenoids, and cold storage ensured their higher retention, which is comparable to the findings from the present study.

The content of β-carotene ranged from ca. 3 mg × 100 g d.w.^−1^ to 4 mg × 100 g d.w.^−1^ in BF0R and C. In turn, its content in BA12 was lower and ranged from 2.3 mg to 3.3 mg × 100 g d.w.^−1^. Peas contained less β-carotene. And analogously to beans, smaller β-carotene losses were noted in the freeze-dried samples than the air-dried samples. The content of this compound decreased throughout storage, and storing the dried material at room temperature contributed to greater losses than in the cold-stored samples; this dependency was observed regardless of the drying method and vegetable species used (Figure 1k). Over the study period, the content of β-carotene decreased by 36% in PF and by 40% in PA (Figure 1k). Other authors have also confirmed the higher retention of this compound during cold storage than during storage under room temperature conditions and its successive decrease along with storage time [50,51,56]. The higher retention of β-carotene in dried materials may also be affected by using airtight and light-proof packages and inner gas (nitrogen) [43].

The retention of total polyphenols was higher during freeze-drying than air drying, regardless of vegetable species. The storage of the dried materials at room temperature caused a greater decrease in the total polyphenol content compared to the cold storage (Figure 1l). Over the 12-month storage, total polyphenol losses exceeded 20% in beans, compared to 11–14% losses noted in peas (Figure 1l). The freeze-dried samples had a significantly higher content of these compounds than the air-dried ones, which is consistent with findings reported by other authors [18,57,58]. Also, Maurya et al. [59] have confirmed higher retention of polyphenols in the freeze-dried samples of bell peppers and lower retention in the dried materials obtained via microwave–vacuum drying, convection drying, and those dehydrated in the sun.

One of the biggest challenges in food science is maintaining the highest antioxidant activity [60]. Air-drying contributed to greater suppression of the antioxidative activity against the DPPH radical than material freeze-drying. The antioxidative activity of dried materials was observed to successively decrease over the storage period, with greater suppression noted in the materials stored at room temperature (Figure 1m). Throughout the experiment, the antioxidative activity against the DPPH radical decreased by over 30% in dried beans and by 40% in dried peas.

#### 2.2.3. Sensory Assessment

When choosing a product, the consumer is guided primarily by sensory impressions and firstly pays attention to color, then consistency, and tastiness. Therefore, selecting the appropriate raw material, pre-treatment, drying method, packaging, and storage conditions is essential to obtain the highest organoleptic quality product that is attractive to a potential buyer. Sensory assessment allows for establishing the extent of changes in the evaluated descriptors during raw material processing and final product storage [61]. In the 5-point assessment method, the highest score was given by panelists to PF12C and R (4.6 points), followed by BF12C and R (4.5 points). Also, PA12C received a high score (4.2 points), followed by PA12R (3.9 points) and BA12C and R received 3.8 and 3.6 points, respectively.

**Figure 2:**
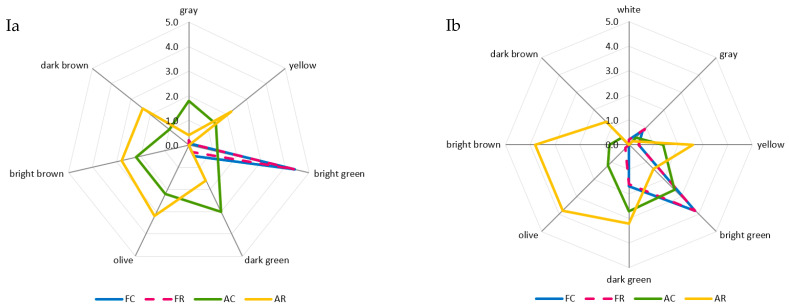

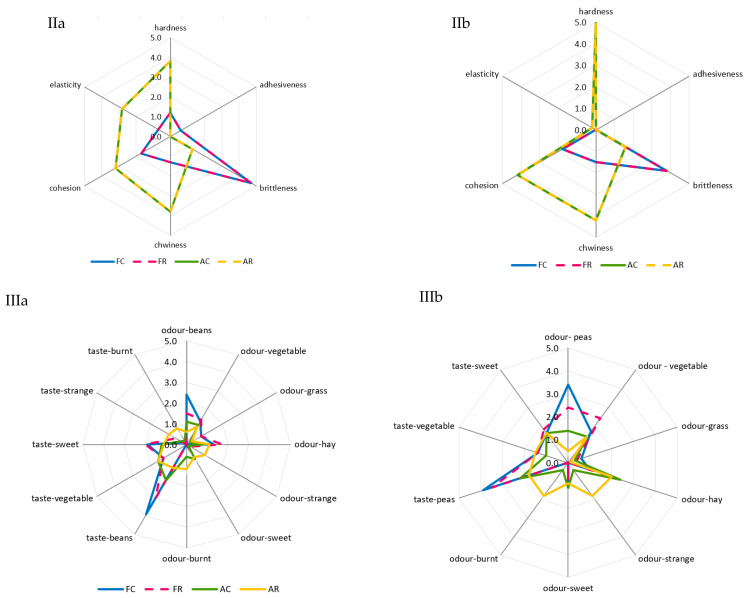
A comparative analysis of: color profile (**I**), texture (**II**), and palatability (**III**) of dried materials from green beans (**a**) and green peas (**b**) obtained via freeze-drying (F) and air-drying (A) and stored under cold storage (C) and room temperature (R) conditions.

The greatest and most diversified changes in color were noted in the case of the air-dried samples. A comparison of room and refrigerated temperatures during BA storage showed that gray and light-green colors disappeared, and the share of dark-green color decreased from moderately to slightly noticeable, whereas the intensity of yellow and dark-brown colors increased from barely to slightly noticeable, and olive and light-brown colors increased from slightly to moderately noticeable (Figure 2Ia). The prevailing colors of PA12C were light-green and dark-green, which in the samples stored at room temperature, were replaced by strongly noticeable olive and light-brown colors and moderately noticeable yellow and barely noticeable dark-brown colors (Figure 2Ib). The color changes of air-dried materials subjected to long-term storage are mainly due to the NEB reactions and water activity that promote these reactions, which, in the case of the analyzed air-dried vegetable sample, ranged from 0.55 to 0.67. These effects were indicated by the successively progressing material darkening and the appearance of brown hues, as well as confirmed by instrumental color measurements and quantitative analysis of pigments, which indicated that the disappearance of green chlorophyll somehow exposed yellow and orange carotenoids. El-Hamzy and Ashour [38] demonstrated that color changes progressed in time, regardless of the drying method applied, when they stored dried ball pepper samples at room temperature for 12 months in polyethylene bags. In addition, after these 12 months, they noted the greatest values for NEB reactions in the dried samples dehydrated in the sun, followed by the samples dried in a dryer at 60 °C, and then those dried in a “refractance window” (RW)-type dryer. The texture of dried materials is affected by both temperature and duration of blanching and drying. The impact of high temperature during pre-treatment triggers significant changes in the mechanical properties of the dried tissue, observed mainly after its hydration and resulting in texture weakening [62]. These changes are due to pectin methyl esterase (PME). This enzyme is responsible for changes in the rigidity of cell walls, which can be activated or deactivated depending on the process temperature applied. At temperatures of 50–70 °C, PME is active and reacts with pectins, enabling the disintegration of intercellular substances, thereby affecting the mechanical and structural properties, density, hardness, porosity, and shrinkage of the material subjected later to the drying process. At temperatures exceeding 70 °C, PME is inactivated, which causes the migration of intercellular substances across the dehydrated material and, ultimately, the weakening of its structure [63,64]. The prevailing textural feature of the freeze-dried samples was brittleness (assessed from strong to very strong), and the panelists also noted a lack of elasticity. The air-dried samples received high scores for hardness, chewability, and cohesiveness, low scores for brittleness and elasticity, and finally showed no adhesiveness. After the 12-month storage of the products, it was found that the storage temperature did not affect the changes in the texture profile either in the freeze-dried or the air-dried samples (Figure 2IIa,b). The palatability of dried vegetables includes taste and aroma. In general, the drying process and storage conditions contributed to the weakening of the taste and aroma typical of a given vegetable species and also resulted in the development of undesirable taste and aroma notes, described as burnt, foreign, hay, and grassy. Guiné [65] reported that the process temperature and the presence of air influenced the losses of volatile compounds and that the lowest possible temperatures and vacuum should be applied to maintain their highest levels. After 12 months of storage, the taste and aroma of beans were still distinct, and foreign aromas (mainly of hay and grass) were also perceptible (Figure 2IIIa). In the case of peas, the taste and aroma typical of this material were more intensively perceptible in the freeze-dried samples. In contrast, the aroma of hay was more intense in the air-dried samples. Moreover, a burnt aroma was more distinct in the air-dried samples of peas stored under room temperature conditions (Figure 2IIIb). The dry hay aroma in peas may be attributed to 5- or 6-methyl-3-isopropyl-2-methoxypyrazine [66,67]. Hoffmann [68] confirmed the presence of hay aroma and also identified a note of dust in the freeze-dried and air-dried dill samples. After drying, the intensity of the above descriptors increased. They were more perceptible in the freeze-dried than air-dried samples, which disagrees with the observations made in the present study for peas. The burnt and caramel aromas are due to the following aldehydes: 2-methylutanol and 3-methylbutanol. The highest amounts of these aldehydes are produced during drying at 90 °C due to the NEB reactions [69,70]. Jacobsen et al. [66] identified these aldehydes in blanched peas and identified 40 other volatile compounds affecting pea aroma, including monounsaturated six-carbon aldehydes, ketones, alcohols, and their derivatives.

#### 2.2.4. Principal Component Analysis (PCA)

**Figure 3:**
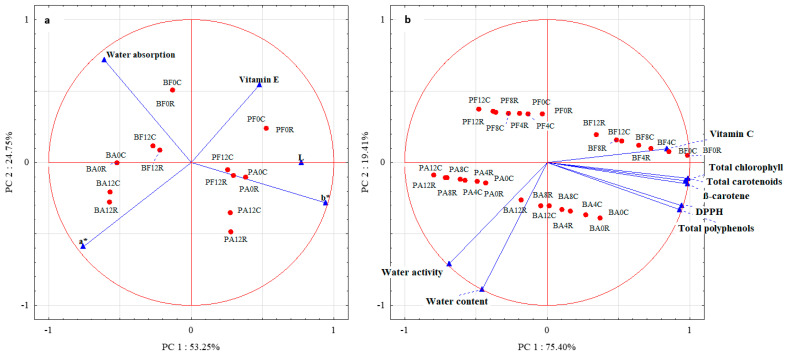
Principal component analysis of dried green beans and peas: (**a**)—between color parameters, water absorption, and vitamin E; (**b**)—between water content and activity, vitamin C, total chlorophylls, total carotenoids, β-carotene, total polyphenols, and antioxidative activity against DPPH. Method of drying (F—freeze drying; A–air-drying), storage period (0, 4, 8, 12 months), and storage temperature (C 2–4 °C; R 18–22 °C).

The PCA results (Figure 3a) demonstrated that the first two components explained 78% of the total variance. The first principal component (PCA1) explained 53.25% of the information, especially in *L*, a*, and b* color parameters. In contrast, the second principal component (PCA2) explained 24.75% of the data variability for water absorption and partly for the a* color parameter and vitamin E content. The analysis of the PCA plot allowed for noticing a strong correlation between PCA1 and the b* color parameter and a relatively strong correlation between *L* and a* color parameters. In turn, the arrangement of vectors between the variables indicates a positive correlation between *L* and b*. A relatively strong correlation was also noticed between water absorption and PCA2, and a medium–strong correlation was noticed between PCA2 and vitamin E content and a*.

The analysis of the distribution of cases against the variables showed an especially high contribution of color parameter b* in PA0C, PA0R, PF12R, and PF12C. Also, *L* was observed to dominate in these vegetable samples. A smaller contribution of the b* parameter was observed in PA12C and PA12R. The contribution of the color parameter a* was high in BA12R and BA12C and slightly smaller in BA0C and R, as well as in BF12C and R. These two types of vegetable samples (BF12R and C) also exhibited a higher water retention capacity than the other samples. A slightly lower water absorption capacity was determined for BF0C and R. In addition, PF0C, R, and PF12C had higher contents of vitamin E.

The PCA results (Figure 3b) demonstrated that the first two components explained 94.81% of the total variance. The PCA1 explained 75.40% of the information provided, especially for the total chlorophyll content, total polyphenol content, total carotenoid content, β-carotene content, antioxidative activity against the DPPH radical, and vitamin C content. In contrast, the PCA2 explained 19.41% of water activity and content data variability. The analysis of the PCA plot demonstrated a strong correlation between PCA1 and total chlorophyll content, total polyphenol content, total carotenoid content, β-carotene content, antioxidative activity against the DPPH radical, and vitamin C content. In turn, the distribution of vectors among the variables indicates a positive correlation between them. A strong correlation was also noted between water content and PCA2, and a relatively strong correlation was found between PCA2 and water activity. The vector arrangement between these variables also indicates a positive correlation between them.

The analysis of the distribution of cases against the variables showed a high vitamin C content, especially in BF4C and R and BF8C, a slightly lower one in BF8R, and also in BF12C and R. All these vegetable samples, and additionally, BF0C and R, had higher contents of total chlorophyll, total carotenoids, and β-carotene compared to the other analyzed samples. A high water content was determined in BA12R, BA8R, and BA12C. In addition, BA12R exhibited high water activity.

Other authors have also demonstrated the impact of the drying method and storage conditions on the contents of vitamin C [18], carotenoids [55], chlorophyll, and β-carotene [51].

## 3. Materials and Methods

### 3.1. Experimental Material

The experimental material included green beans and green peas. The legumes were grown at the experimental plot of the Department of Fruit, Vegetables and Mushrooms Technology of the Kraków Agricultural University in the framework of a research project of the Ministry of Science and Higher Education in Poland, no. NN312 441837.

**Figure 4:**
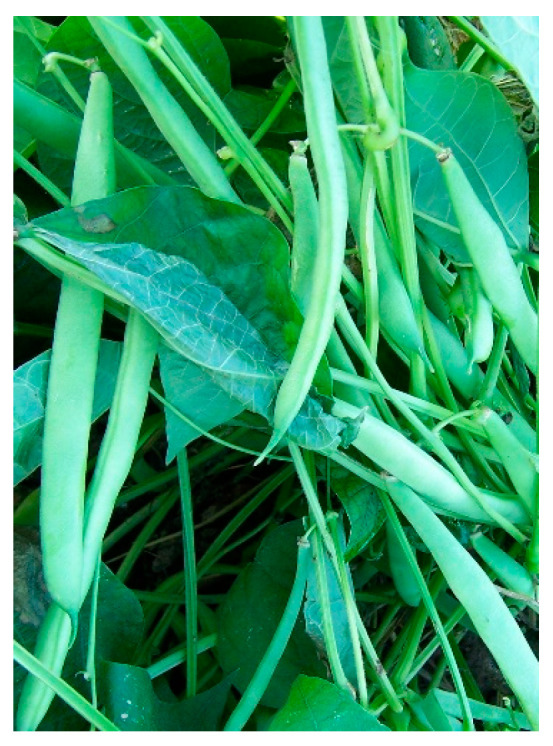
*Phaseolus vulgaris* L.

Green beans (*Phaseolus vulgaris* L.), as seen in Figure 4, are of the ‘Gracja’ cultivar, produced by the Polan company (Poland). The growing season from sowing until the harvest of green pods spanned 80–90 days. The pods were green, 11–13 cm long, 0.8–0.9 cm wide, round in cross-section, stringless, straight in shape, and set in the central part of the plant. Properly developed pods were harvested at the end of July, during rainless weather.

**Figure 5:**
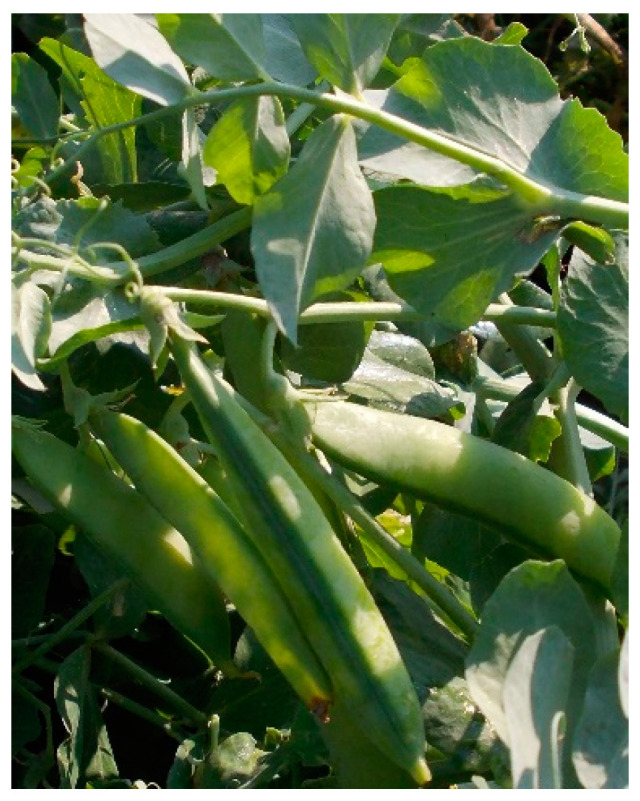
*Pisum sativum* L.

Green peas (*Pisum sativum* L.), as seen in Figure 5, are of the ‘Cud Kelvedonu’ cultivar, produced by the Torseed company (Poland). They are classified as an early, dwarf, or medium-tall cultivar. They consist of a shell pea variety grown for seeds. The pods were collected when the seeds were large and green before reaching the physiological maturity phase (immature). One pod produced 6–7 seeds. The growing season from sowing till the green seed harvest spanned approximately 80 days. The pods were collected at the beginning of July, i.e., at the optimal dry matter content of the seeds.

Table 1 and Supplementary Figure S1 present the characteristics of raw materials and the effects of their pre-treatment.

### 3.2. Production of Dried Materials

**Figure 6:**
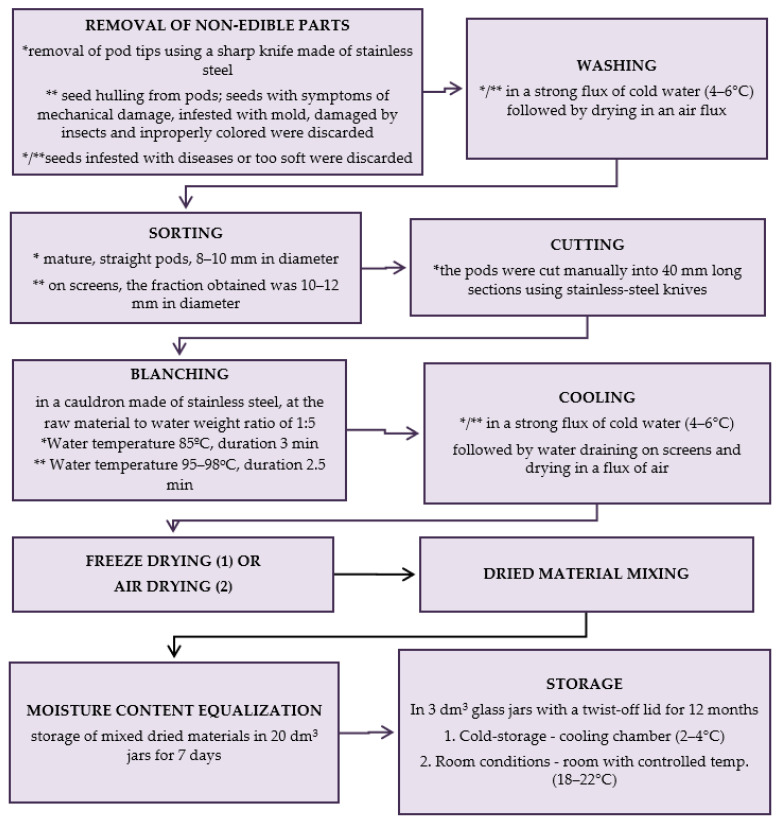
Scheme of production of dried materials; * green beans, ** green peas; methods (1) and (2) are described below. (1) Freeze-drying; firstly, the vegetables were arranged in a single layer on trays of a freeze-dryer (vegetable load per 1 m² of trays was 7.5 kg) and frozen at a temperature of −40 °C in a Feutron 3626-51 freezing chamber (Germany). The time required to reach −30 °C inside the frozen vegetables was 90 min. The frozen vegetables were freeze-dried in a Gamma 1–16 LSC freeze-dryer (Christ, Germany) at the following conditions: 1. initial drying: temp. of frozen material (−30 °C); temp. of a condenser (−52 °C); temp. of shelves (+20 °C); 2. additional drying: the last approx. 6 h of the total drying time (temp. of shelves +30 °C). Drying time at a shelf load of 7.5 kg × m^−2^ to the water content < 3% assumed in Methods; * green beans—28 h, ** green peas—30 h; (2) Air-drying; air drying was done using electric air dryers (Zorpot Zalmet, Poland). The vegetable load was 8.5 kg × m^−2^ screen surface area. The inlet hot air temperature was 60 °C. Drying was continued until the dried vegetables had 10% moisture content. * green beans—8 h, ** green peas—9 h.

The production scheme of dried materials from raw material collection till storage is presented in Figure 6.

**Figure 7:**
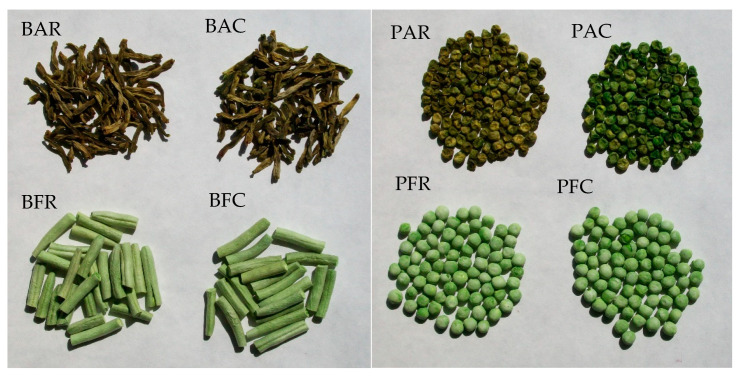
Dried green beans (B) and green peas (P) after twelve months of storage; A—air-dried, F—freeze-dried, R—room storage, C—cooling storage.

Contents of water, vitamin C, total chlorophyll, total carotenoids, β-carotene, and total polyphenols, as well as antioxidative activity against the DPPH radical, were determined in raw materials, blanched materials, dried materials immediately after drying (0 months), and dried materials after 4, 8, and 12 months of cold storage (2–4 °C) and storage at room temperature (18–22 °C) (Figure 7). Color assessment (parameters *L*, a*, b*) and vitamin E content determination were performed in raw materials, blanched materials, dried materials immediately after drying, and dried materials after 12 months of cold storage and storage at room temperature. The dried vegetables were determined for water activity (a_w_) at each stage of storage; in turn, their water absorption capacity was determined immediately after drying and after 12 months of storage, whereas the sensory assessment was made only for the 12-month-stored dried vegetables.

### 3.3. Methods

#### 3.3.1. Physical Analyses

##### Preparation of Raw Material for Analyses

Fresh vegetables were homogenized with deionized water at a 1:1 ratio, whereas the dried ones were homogenized at a 1:19 ratio in a Microtron MB 550 high-speed laboratory homogenizer (Kinematica AG, Malters, Switzerland).

##### Determination of Water Content

Water content of the samples was determined with the gravimetric method according to the AOAC standard [71]. A ca. 10 g sample of pulp (prepared as described in Section Preparation of Raw Material for Analyses) was weighed into measuring vessels, pre-dried on an electric cooktop, and then dried to a constant weight at temperatures of 98–100 °C in a Heraeus instrument T12 laboratory dryer (Hanau, Germany).

##### Determination of Water Absorption Capacity

Dried vegetables’ water absorption capacity was determined following a Polish Standard method [72]. To this end, 10 g of dried vegetables were weighed in a 400 mL beaker. Next, 250 mL of distilled water was added to the beaker, closed tightly with an aluminum foil cap, and left at a temp. of ca. 20 °C for 24 h. Afterwards, the content of the beaker was filtered through a fluted filter (12.5 cm in diameter) into a dry 250 mL cylinder. During filtration, the funnel with the filter was covered with a watch glass. The fluid’s volume was read out after 60 min of filtration. The water absorption was computed from a difference of water volume used and filtrate volume, considering the amount of filtrate left on the filter.

##### Determination of Water Activity

Water activity of the samples was measured in an AquaLab Series 4TE apparatus (Decagon Devices, Pullman, WA, USA), operating based on dew point detection. The analyzed sample was placed in a plastic vessel (40 mm in diameter and 12 mm in height), which was then located in an air-tight measuring chamber of the apparatus. Water activity was determined at 25 °C.

##### Color Assessment with the Instrumental Method

Homogenized dried vegetables (prepared as described in Section Preparation of Raw Material for Analyses) were placed on Petri dishes, frozen at a temperature of −40 °C (Feutron 3626–51, Langenwetzendorf, Germany), and then freeze-dried (Gamma 1-16 LSC, Christ, Osterode am Harz, Germany). Thus, obtained lyophilizates were ground in a mortar to achieve powder with a homogenous consistency.

Instrumental color measurement in the CIELab scale was performed using a MINOLTA CM-3500d multi-functional spectrophotometer with SpectraMagic Color NX software ver. 1.9 for data collection and processing (Konica Minolta, Osaka, Japan). Color measurements were conducted via the reflection method using D65 illuminant with an 8 mm observer, at the measurement angle of 10 °. The material was placed in glass cuvettes (34 mm in diameter and 25 mm in height), and the thickness of the analyzed material in the cuvette was 20 mm.

#### 3.3.2. Chromatographic Analyses

Chromatographic analyses were performed using a Merck HITACHI system consisting of an on-line degasser, autosampler, pump, fluorescence detector, and a column thermostat with D-7000 HPLC-System-Manager software v. 4.1 (HSM).

##### Determination of Vitamin C Content

Vitamin C content was determined via high performance liquid chromatography (HPLC) according to the European Standard [73], using an Onyx Monolithic C18 column with a Guard Cartridges 5 × 4.6 mm pre-column (both from Phenomenex, Torrance, CA, USA) at the wavelength of l = 254 nm. To this end, 0.1% metaphosphoric acid (*v*/*v*) was used as the mobile phase at the isocratic flow rate of 0.9 mL × min^−1^. The total content of L-ascorbic acid and L-dehydroascorbic acid was determined after reduction with an L-cysteine solution. Vitamin C was identified and quantified using an internal standard, i.e., L-ascorbic acid dissolved in 20% metaphosphoric acid (*w*/*v*), with concentrations of 1.0–50 µg × mL^−1^.

##### Determination of Vitamin E Content

Vitamin E content was determined as the sum of α-, β-, γ-, and δ-tocopherols, with the high-performance liquid chromatography (HPLC) method according to the European Standard [74] and the Katsanidis and Addis [75] method with their own modifications. The analysis was conducted on the freeze-dried samples using a LUNA 5μm NH2 250 × 4.5 nm chromatographic column (Phenomenex, Torrance, USA) at the excitation and emission wavelengths of 290/330 nm. The mobile phase was made of *n*-hexane with 2-propanol mixed in a 95% to 5% ratio, applied in the isocratic elution at 2.5 mL × min^−1^. The column was thermostated at a temperature of 30 °C. Identification and quantification of α-, β-, γ-, and δ-tocopherols (Supplementary Figure S2) were conducted using external standards dissolved in *n*-hexane with concentrations in the range of 0.5–2.0 μg × mL^−1^. Vitamin E activity was calculated as α-tocopherol equivalents (α-TE) per 100 g of vegetable according to the equation:

Vitamin E activity=1×α-tocopherol+0.5×β-tocopherol+0.1×γ-tocopherol+0.03×δ-tocopherol

#### 3.3.3. Spectrophotometric Analyses

Spectrophotometric analyses were conducted using a Shimadzu UV-VIS type 160A double-beam spectrophotometer (Shimadzu Europe Gmbh, Duisburg, Germany).

##### Determination of Contents of Total Chlorophylls and Total Carotenoids

The contents of total chlorophylls and total carotenoids were determined with the method provided by Lichtenthaler and Buschmann [76]. To this end, samples of the fresh or dried material were ground with sand in a porcelain mortar with a small amount of pure and cold acetone. The resulting extract was decanted and filtrated to a measuring flask. The grinding was repeated 2–3 times until complete sample discoloration. Next, extract absorbance was measured against pure acetone at the following wavelengths: 470 nm, 661.6 nm, and 644.8 nm, at the absorption maxima of carotenoids, chlorophyll_a_, and chlorophyll_b_. The sample’s concentration of chlorophylls and carotenoids was computed using specific absorption coefficients determined for carotenoids and chlorophylls at the above-mentioned wavelengths.

$${Chlorophyll}_{a}\;[\frac{\mu g}{mL}]=11.24\times A_{661.6}-2.04\times A_{644.8}$$

$${Chlorophyll}_{b}\;[\frac{\mu g}{mL}]=20.13\times A_{644.8}-4.19\times A_{661.6}$$

$$\begin{array}{l} Total\;carotenoids\;[\frac{\mu g}{mL}]\\ =\frac{1000\;\times \;A_{470}\;-\;1.9\;\times \;{Chlorophyll}_{a}\;-\;{63.14\;\times \;Chlorophyll}_{b}}{214} \end{array}$$

##### Determination of β-Carotene Content

The content of β-carotene was determined with the column chromatography method according to the Polish Standard [77]. The method consisted of isolating carotenoids extracted from the analyzed sample, separating them on a chromatographic column, and spectrophotometrically determining the β-carotene content. β-Carotene was eluted first among the carotenoids on the chromatographic column. The absorbance of the β-carotene-containing fraction was measured at 450 nm. The content of β-carotene was computed using a standard curve plotted for a potassium dichromate solution.

##### Determination of Total Polyphenol Content

The total polyphenol content was determined via the Folin–Ciocalteau method [78]. To this end, a sample was weighed from the obtained homogenate (as described in Section Preparation of Raw Material for Analyses), from which polyphenols were extracted with an 80% (*v*/*v*) aqueous methanol solution using a DI 25 Basic homogenizer (IKA Works, Inc., Wilmington, NC, USA). The samples were centrifuged at 12,000 rpm for 10 min, at a temperature of 4 °C (MPW-260R centrifuge, MPW Med. Instruments, Warsaw, Poland), and then filtered using Whatman No. 4 filter paper into a measuring flask that was filled up with 80% (*v*/*v*) methanol.

Then, the Folin–Ciocalteau reagent and a 25% (*w*/*v*) aqueous solution of sodium carbonate were added to an appropriate amount of the supernatant. Absorbance was measured after 60 min at the wavelength of 675 nm. The content of total polyphenols was read out from a standard curve plotted for (+)-catechin.

##### Determination of Antioxidative Activity against the DPPH Radical

The antioxidative activity was determined with a method using a free DPPH (1,1-diphenyl-2-picrylhydrazyl) radical according to Pekkarinen et al. [79]. After homogenization with water (as described in Section Preparation of Raw Material for Analyses), the samples were hot-extracted with 80% (*v*/*v*) ethanol. After cooling, they were centrifuged at 4500 rpm at a temperature of 4 °C (MPW-260R centrifuge, MPW Med. Instruments, Warsaw, Poland). The antioxidative activity was determined by evaluating changes in the absorbance of the samples measured 0 and 10 min after a free radical solution addition to the supernatant. Absorbance was measured at the wavelength of 516 nm and expressed in µmol Trolox equivalents/g of product dry matter.

#### 3.3.4. Sensory Assessment

The air-dried and freeze-dried materials were evaluated after 12 months of cold storage and storage at room temperature. Four methods were deployed in the assessment, i.e., the direct method using a 5-point evaluation scale [80] and auxiliary methods involving color profiling, texture profiling, and palatability profiling. The 5-point assessment was performed under conditions consistent with the Polish Standard [81] by a group of panelists meeting basic requirements in terms of sensory sensitivity, as set in the Polish Standard [82]. The assessment included basic quality attributes. The overall sensory quality indicator was calculated using weight coefficients determined for individual product quality attributes. The color assessment of the vegetable dried materials was conducted with the quantitative descriptive analysis (QDA) method according to the International Standard [83]. In contrast, texture analysis was performed with the QDA method according to the Polish Standard [84], which consisted of the evaluation of the mechanical parameters of products, including hardness, cohesiveness, elasticity, and adhesiveness, as well as brittleness and chewiness. Palatability assessment of the dried vegetable materials was conducted with the QDA method according to the Polish Standard [85] by identifying aroma and taste features (notes), determining product palatability, and determining their intensity.

To specify the intensity of individual color, mechanical, and palatability features of the analyzed products, each feature was evaluated on a scale from 0—‘imperceptible feature’ to 5—‘very noticeable feature’.

#### 3.3.5. Statistical Analysis

All examinations were performed in triplicate (*n* = 3). The results obtained were subjected to statistical analysis using STATISTICA 13.3 software (Statsoft, Inc., Tulsa, OK, USA) and expressed as mean ± standard error of the mean. One-way or multi-way analysis of variance was performed. The significance of differences between the means was estimated on the basis of Tukey’s post hoc test at *p* < 0.05. In addition, Principal Components Analysis (PCA) was conducted to establish the effect of vegetable species, drying methods, storage conditions, and storage period on the physical properties and contents of bioactive components in the dried materials.

## 4. Conclusions

The legumes, particularly beans and peas, can be processed into high-quality dried materials. The drying process performed in the present study reduced water activity to a value that ensured microbiological safety throughout storage. Both drying and storage reduced the content of vitamin C and, to a lesser extent, the content of other bioactive components. The freeze-dried samples were characterized by higher retention of the analyzed antioxidants except for chlorophyll, lower water activity, and higher rehydration capacity than the air-dried products. The storage temperature significantly impacted the results obtained, and the storage of dried vegetables in refrigerated conditions promoted the retention of bioactive components.

The strength of this research is in its extensive analysis of dried vegetables in terms of changes in the contents of selected bioactive components, as well as their physical and sensory features not only after the dehydration process but also after long-term storage. Moreover, the experimental setup was planned in such a way as to best reflect the conditions of everyday use in households or gastronomy. This means that the dried vegetables were stored without access to light in airtight vessels, without a modified atmosphere, and opened during the collection of material for analyses, as is most often the case when consumers handle them.

A drawback of the research is the lack of instrumental texture analysis, which would make it possible to more objectively illustrate changes occurring in the finished product that affect consumer acceptability. These analyses are planned for our future investigations.

## Figures and Tables

**Table 1:** The effect of vegetable and pre-treatment on the least-squares mean of the selected physical parameters and contents of bioactive components in the analyzed materials (mean ± SE).

	Water Content [g]	*L*	a*	b*	Vitamin C [mg]	Vitamin E [mg]	Total Chlorophyll [mg]	Total Carotenoid [mg]	β-Carotene [mg]	Total Polyphenols [mg (+) Catechin]	DPPH [μmol Trolox Equivalent]
	100 g f.w.				100 g d.w.						1 g d.w.
Vegetable											
Green beans	91.26 ± 0.13 ^b^	55.81 ± 1.48 ^a^	−8.98 ± 0.48 ^a^	17.24 ± 0.54 ^a^	174.63 ± 5.89 ^b^	4.07 ± 0.04 ^a^	96.63 ± 2.87 ^b^	15.39 ± 0.10 ^b^	4.30 ± 0.05 ^b^	532.13 ± 6.98 ^b^	91.88 ± 1.20 ^b^
Green peas	81.02 ± 0.22 ^a^	71.07 ± 1.47 ^b^	−9.43 ± 0.21 ^a^	29.01 ± 0.60 ^b^	151.38 ± 5.51 ^a^	5.04 ± 0.05 ^b^	35.25 ± 0.87 ^a^	10.26 ± 0.11 ^a^	2.50 ± 0.03 ^a^	290.25 ± 4.04 ^a^	36.88 ± 1.17 ^a^
Pre-treatment											
Raw	86.15 ± 1.98 ^a^	66.01 ± 2.75 ^b^	−10.05 ± 0.2 ^b^	22.20 ± 2.29 ^a^	177.25 ± 5.14 ^b^	4.64 ± 0.19 ^b^	70.41 ± 12.56 ^b^	12.79 ± 0.96 ^a^	3.44 ± 0.35 ^a^	406.75 ± 42.38 ^a^	67.00 ± 10.41 ^b^
Blanched	86.13 ± 1.91 ^a^	60.87 ± 3.40 ^a^	−8.36 ± 0.21 ^a^	24.05 ± 2.25 ^b^	149.75 ± 4.47 ^a^	4.47 ± 0.17 ^a^	61.46 ± 10.70 ^a^	12.86 ± 0.99 ^a^	3.36 ± 0.34 ^a^	415.63 ± 49.42 ^a^	61.75 ± 10.41 ^a^

Different letters in columns for the given attribute denote statistically significant differences at *p* < 0.05, d.w.—dry weight, f.w.—fresh weight.

**Table 2:** The least-squares mean of water content, a_w_, vitamin C content, total chlorophyll content, total carotenoid content, total polyphenol content, and antioxidant activity against the DPPH radical, depending on the vegetable species, drying method, storage time, and storage conditions. Mean ± SE.

	Water Content [g]	a_w_	Vitamin C [mg]	Total Chlorophyll [mg]	Total Carotenoid [mg]	β-Carotene [mg]	Total Polyphenols [mg (+) Catechin]	DPPH [μmol Trolox Equivalent]
	100 g f.w.		100 g d.w.					1 g d.w.
Vegetable								
Green beans	6.36 ± 0.43 ^a^	0.317 ± 0.032 ^a^	109.23 ± 3.23 ^b^	67.02 ± 1.77 ^b^	12.38 ± 0.20 ^b^	3.25 ± 0.06 ^b^	463.78 ± 5.91 ^b^	71.86 ± 1.39 ^b^
Green peas	6.37 ± 0.45 ^a^	0.461 ± 0.026 ^b^	83.56 ± 2.95 ^a^	26.10 ± 0.46 ^a^	8.27 ± 0.13 ^a^	1.81 ± 0.05 ^a^	252.56 ± 2.17 ^a^	23.25 ± 0.68 ^a^
Drying method								
Freez-dried	2.87 ± 0.02 ^a^	0.160 ± 0.012 ^a^	108.41 ± 3.54 ^b^	54.14 ± 3.24 ^b^	11.16 ± 0.34 ^b^	2.79 ± 0.11 ^b^	373.41 ± 14.53 ^b^	51.84 ± 3.32 ^b^
Air-dried	9.87 ± 0.02 ^b^	0.618 ± 0.007 ^b^	84.39 ± 2.69 ^a^	38.98 ± 2.08 ^a^	9.48 ± 0.23 ^a^	2.27 ± 0.09 ^a^	342.94 ± 13.24 ^a^	43.28 ± 3.10 ^a^
Storage period [month]								
0	6.30 ± 0.63 ^a^	0.383 ± 0.043 ^a^	124.06 ± 4.16 ^d^	52.29 ± 4.50 ^d^	11.48 ± 0.43 ^d^	2.93 ± 0.15 ^d^	391.69 ± 22.98 ^d^	56.13 ± 5.02 ^d^
4	6.36 ± 0.63 ^ab^	0.386 ± 0.043 ^a^	105.34 ± 3.61 ^c^	47.58 ± 4.16 ^c^	10.76 ± 0.41 ^c^	2.72 ± 0.14 ^c^	370.06 ± 20.46 ^c^	50.44 ± 4.72 ^c^
8	6.40 ± 0.63 ^b^	0.392 ± 0.044 ^a^	82.56 ± 3.09 ^b^	44.83 ± 3.95 ^b^	9.93 ± 0.39 ^b^	2.35 ± 0.14 ^b^	343.28 ± 17.06 ^b^	44.03 ± 4.28 ^b^
12	6.43 ± 0.63 ^b^	0.395 ± 0.044 ^a^	73.63 ± 3.01 ^a^	41.52 ± 3.53 ^a^	9.13 ± 0.42 ^a^	2.12 ± 0.14 ^a^	327.66 ± 16.87 ^a^	39.66 ± 3.90 ^a^
Storage temperature [°C]								
2–4	6.36 ± 0.44 ^a^	0.387 ± 0.030 ^a^	101.47 ± 3.29 ^b^	48.00 ± 2.91 ^b^	10.55 ± 0.30 ^b^	2.61 ± 0.10 ^b^	364.03 ± 14.33 ^b^	49.77 ± 3.32 ^b^
18–22	6.38 ± 0.44 ^a^	0.391 ± 0.031 ^a^	91.33 ± 3.57 ^a^	45.11 ± 2.85 ^a^	10.09 ± 0.31 ^a^	2.45 ± 0.11 ^a^	352.31 ± 13.68 ^a^	45.36 ± 3.16 ^a^

Different letters in columns for the given attribute denote statistically significant differences at *p* < 0.05, d.w.—dry weight, f.w.—fresh weight.

**Table 3:** Least-squares mean of water absorption, color parameters, and vitamin E content, depending on the vegetable species, drying method, and storage time and conditions, mean ± SE.

	Water Absorption [mL]	*L*	a*	b*	Vitamin E [mg]
	100 g d.w				100 g d.w.
Vegetable					
Green beans	694.69 ± 25.24 ^b^	63.97 ± 1.50 ^a^	−2.67 ± 0.56 ^a^	3.08 ± 0.48 ^a^	3.00 ± 0.13 ^a^
Green peas	330.53 ± 15.15 ^a^	74.19 ± 0.58 ^b^	−5.92 ± 0.39 ^b^	30.07 ± 0.85 ^b^	3.57 ± 0.18 ^b^
Drying method					
Freez-dried	620.78 ± 38.07 ^b^	73.41 ± 0.84 ^b^	−6.68 ± 0.33 ^b^	16.14 ± 2.00 ^a^	3.40 ± 0.19 ^b^
Air-dried	404.44 ± 28.30 ^a^	64.75 ± 1.53 ^a^	−1.90 ± 0.40 ^a^	17.02 ± 2.95 ^b^	3.16 ± 0.15 ^a^
Storage period					
0	534.50 ± 40.45 ^b^	66.63 ± 1.43 ^a^	−5.23 ± 0.55 ^a^	17.63 ± 2.51 ^b^	4.15 ± 0.09 ^b^
12	490.72 ± 36.58 ^a^	71.53 ± 1.36 ^b^	−3.35 ± 0.53 ^b^	15.52 ± 2.52 ^a^	2.41 ± 0.03 ^a^
Storage temperature					
2–4	518.22 ± 38.85 ^b^	68.45 ± 1.39 ^a^	−4.50 ± 0.56 ^b^	16.24 ± 2.50 ^b^	3.30 ± 0.16 ^b^
18–22	507.00 ± 38.65 ^a^	69.72 ± 1.51 ^b^	−4.08 ± 0.57 ^a^	16.92 ± 2.58 ^a^	3.26 ± 0.17 ^a^

Different index letter in columns for the given attribute denote statistically significant differences at *p* < 0.05; d.w.—dry weight.

## Data Availability

The raw data supporting the conclusions of this article will be made available by the authors upon request.

## Supplementary Materials

The following supporting information can be downloaded at: https://www.mdpi.com/article/10.3390/molecules29081732/s1. Figure S1: The effect of blanched on water content (in 100 g f.w), color parameters, contents of selected bioactive components (in 100 g d.w.) and antioxidant properties (in 1 g d.w.) in the green peas and green beans (mean ± SE). Different letters denote statistically significant differences at *p* < 0.05, d.w.—dry weight, f.w.—fresh weight. Figure S2: The chromatogram of tocopherols in raw green peas; retention time: 4 min—α-tocopherol, 10 min—β-tocopherol, 11 min—γ-tocopherol.

## Abbreviations

vegetable	(B—dried green beans; P—dried green peas)
method of drying	(F—freeze-drying; A—air—drying),
storage of period	(0, 4, 8, 12 months)
temperature of storage	(C—cooling storage 2–4 °C; R—room storage 18–22 °C)
NEB	non-enzymatic browning
PME	pectin methylesterase
