# Supplementary material for: Effect of the Drying Method and Storage Conditions on the Quality and Content of Selected Bioactive Compounds of Green Legume Vegetables
Source: Molecules. 2024 Apr 11;29(8):1732. doi: 10.3390/molecules29081732 (PMC11052391; doi:10.3390/molecules29081732)
Supplement: Supplementary file 1 [file molecules-29-01732-s001.zip › Supp1.pdf]

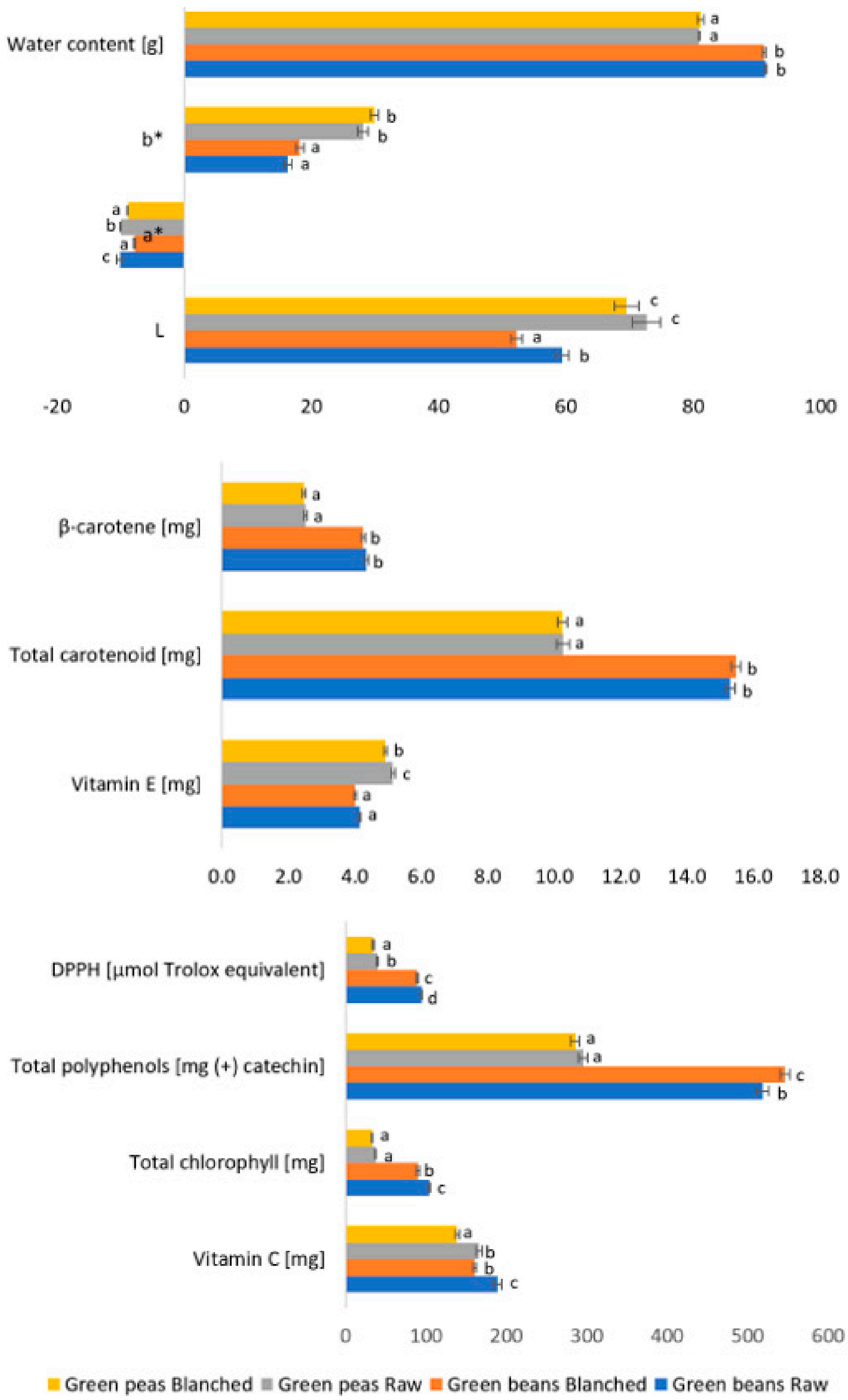

Figure S1. The effect of blanched on water content (in 100 g f.w) , color parameters, contents of selected bioactive components (in 100 g d.w.) and antioxidant properties (in 1 g d.w.) in the green peas and green beans (mean±SE). Different letters denote statistically significant differences at $p<0.05$, d.w. – dry weight, f.w. – fresh weight
