# Supplementary material for: Effect of the Drying Method and Storage Conditions on the Quality and Content of Selected Bioactive Compounds of Green Legume Vegetables
Source: Molecules. 2024 Apr 11;29(8):1732. doi: 10.3390/molecules29081732 (PMC11052391; doi:10.3390/molecules29081732)
Supplement: Supplementary file 1 [file molecules-29-01732-s001.zip › Supp2.pdf]

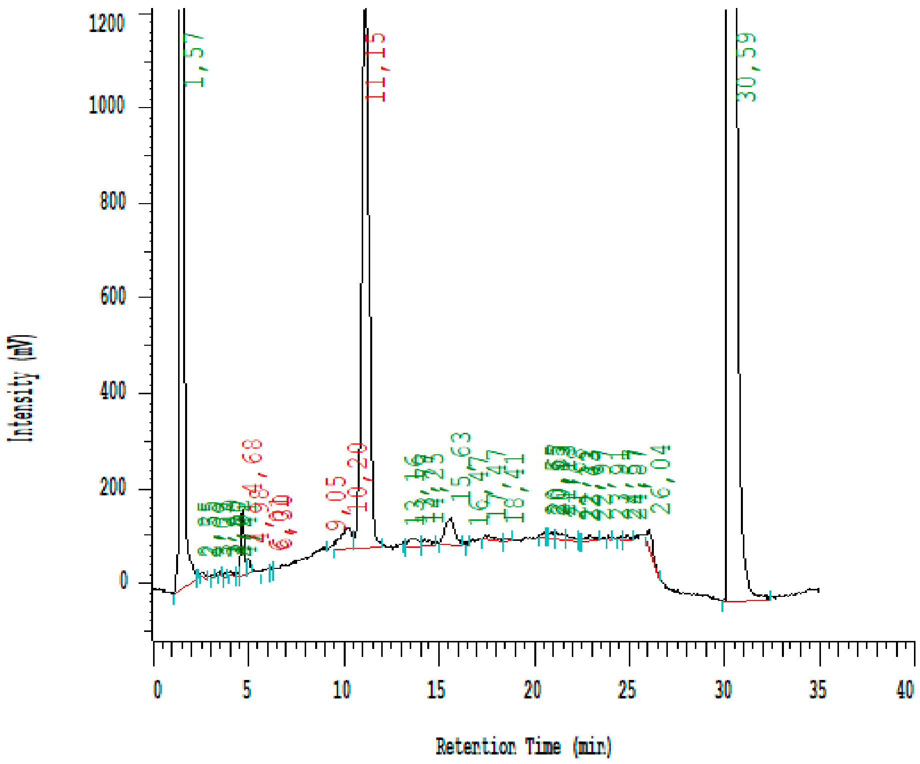

Figure S2. The chromatogram of tocopherols in raw green peas; retention time: 4 min – α-tocopherol, 10 min – β-tocopherol, 11 min – γ-tocopherol.
